# Analysis and effect of the use of biofertilizers on *Trifolium rubens* L., a preferential attention species in Castile and Leon, Spain, with the aim of increasing the plants conservation status

**DOI:** 10.3934/microbiol.2017.4.733

**Published:** 2017-08-22

**Authors:** Xavier Cruz-González, Nereha Laza-Pérez, Pedro F. Mateos, Raúl Rivas

**Affiliations:** 1 Department of Microbiology and Genetics, University of Salamanca, Salamanca, Castile & Leon, Spain; 2 Spanish-Portuguese Institute for Agricultural Research (CIALE), Salamanca, Castile & Leon, Spain; 3 Associated I + D Unit, USAL-CSIC (IRNASA), Salamanca, Castile & Leon, Spain

**Keywords:** *Trifolium rubens* L., preferential attention plant, *Rhizobium*, PGPR, nodulation

## Abstract

*Trifolium rubens* L. is a leguminous plant “Preferential Attention”, according to the Catalog of Protected Flora of Castile and Leon (Spain). In this study we aimed to analyze the potential of three bacterial strains of the genus *Rhizobium* to improve the growth and development of this plant. All three strains produced 3-indoleacetic acid (IAA), but the strain ATCC 14480 produced the most. In addition, all strains produced biofilms and cellulases, although in different quantities. The synthesis of these products has been described as being related to the processes of the adherence of bacteria to the plant root surface and their entrance into the plant, respectively. In addition, *in vitro* assays and assays conducted under controlled and sterile conditions were performed, showing that the three strains were capable of nodulating *T. rubens* L. and effectively fixed nitrogen for the plant. These results were corroborated by morphological and histological analysis of nodules. Finally, greenhouse assays determined the effects of the strains under more competitive conditions, and it was concluded that inoculated plants presented greater lengths and weights, both aerial and radicular, and also chlorophyll and nitrogen content compared to the uninoculated controls.

## 1. Introduction

The Autonomous Region of Castile and Leon, Spain is a territory rich in vascular flora due to its geographical position. However, the disappearance of both animal and plant species could endanger the survival of others species and consequently alter the delicate ecological balance and interactions [1]. As a preventative measure to protect the plant species within the region, the Decree 63/2007 was created and established the Catalog of Protected Flora of Castile and Leon [2]. This document lists the plants within this area according to their conservation status, where species that require protection are categorized as being endangered and vulnerable. In addition to the most threatened species, this catalog also includes species of special interest, preferential attention and species whose exploitation is regulated. With regard to the category of preferential attention, these species are defined as having reduced populations in areas that are affected by certain events, and therefore require specific management plans; *Trifolium rubens* L. is included within this category.

The genus *Trifolium* is native to Europe, America, and Africa, and is widely distributed in temperate and subtropical regions. This genus includes approximately 250–300 species, which belong to the family Fabaceae, subfamily Papilionoideae [3]–[6]. *Trifolium* was described by Linnaeus in the Species Plantarum, published in 1753, as perennial herbaceous plants with trifoliate leaves and seated leaflets, whose fruit is a legume with a pair of seeds inside [7]. The species within this genus are of great importance to humans as they are often used to improve impoverished soils by replenishing nutrients such as nitrogen. In addition, *Trifolium* serves as food for some animals and can even be used for ornamental purposes.

Despite its use as a source of nitrogen, there are very few studies concerning the symbiotic interaction between *Rhizobium* sp. and *T. rubens* L. Hence, more in-depth knowledge about the interaction of some nitrogen-fixing bacteria, such as *Rhizobium*, in symbiosis with this plant could potentially help to improve the conservation status of this species by promoting plant growth. That is to say, a positive plant-rhizobia interaction may increase the amount of nitrogen fixed exclusively for the plant, which is an essential element in the building of macromolecules such as nucleic acids, amino acids, vitamins, among others. Rhizobia are bacteria which contain nitrogenases that convert diatomic nitrogen (N_2_) to the ammonium ion (NH_4_^+^), an assimilable compound for plants. Therefore, this symbiotic relationship permits the leguminous plant to obtain a source of nitrogen, at the same as providing the bacteria with a source of carbon, vital for its functions [8],[9]. The extent of nodulation varies, and depends on the genetic characteristics of both symbionts: the plant and the rhizobium [10]. The different strains of *Rhizobium* *leguminosarum* bv. trifoli are one of the most effective rhizobia in fixing atmospheric nitrogen in a diverse range of annual clover species [11],[12].

Furthermore, microbial biofertilizers are combinations of one or more microorganisms, which somehow improve the availability of nutrients when applied to crops. Some of the other advantages of the use of biofertilizers include less pollution, a lower reduction of biodiversity, enhanced soil fertilization and fewer production costs [13]. By contrast, the indiscriminate use of agrochemicals causes environmental pollution, reduced biodiversity and adverse health effects [14],[15]. Hence, studies focused on the use of microorganisms to promote plant growth are becoming increasingly important.

As regards, the efficient isolation and genetic characterization of strains, in addition to the analysis of Plant Growth Promoting Rhizobacteria or “PGPR” able to form biofilms, produce cellulases and Indole-3-acetic acid, that efficiently nodulate plants could ultimately be used to improve plant growth and development. Accordingly, in this study, the application of three *Rhizobium* strains on *Trifolium rubens* L. was tested to determine whether these isolates could be applied as biofertilizers, promoting plant growth and improving the species conservation status.

## 2. Materials and Methods

Bacterial strains were isolated from *Trifolium repens* L. in order to test whether they were able to improve the growth of *T. rubens* L. plants. In addition, *Rhizobium leguminosarum* bv. trifolii ATCC 14480, (from collections) a strain isolated from *Trifolium pratense* L. and capable of nodulating various clover species, was also used. Subsequently, their genetic profile was analyzed by RAPD to identify the different strains, and also the 16S rRNA gene was sequenced to determine the genus. Then, their potential as PGPR was evaluated by testing their capacity to produce Indole-3-acetic acid (IAA), which favors the growth and development of both primary and secondary roots and overall nutrient acquisition, to produce exopolysaccharides, such as cellulose, to produce cellulases, and finally their capacity to develop biofilms for the purpose of adhering to the plant root system. It was later assessed whether these strains were able to efficiently nodulate the plants of *T. rubens* L. These experiments were carried out under sterile conditions, where each strain was allowed to interact directly with the plant. Finally, greenhouse assays were performed to check the behavior of the strains under controlled but somewhat more competitive conditions.

### 2.1. Isolation of bacteria endophytes from *Trifolium repens* L.

Two of the three strains used in this study were microorganisms isolated from the interior of nodules of *Trifolium repens* L. plants cultivated in peat soil and grown under greenhouse conditions. After 4 weeks of cultivation, plants were carefully extracted from their pots and the root nodules were excised. These nodules were superficially sterilized with 2.5% of HgCl_2_ for 2 minutes, followed by 5 washes with sterile distilled water. Then, they were smashed and cultured in Yeast Mannitol Agar (YMA) medium (K_2_HPO_4_ 0.2 g/L, MgSO_4_ 0.2 g/L, mannitol 7.0 g/L, yeast extract 2.0 g/L and agar 20.0 g/L) at 28 °C and monitored daily before for endophyte isolation. The two strains isolated were called NTRH2 and NTRH6, and the third strain was *Rhizobium leguminosarum* bv. trifolii (ATCC 14480). All three strains were cryopreserved at –80 °C in 30% glycerol.

### 2.2. DNA extraction and genetic analysis

DNA was obtained from the bacterial suspension using lysis buffer (NaOH 0.2 g, SDS 0.25 g and 100 ml of sterile distilled water). The mixture was heated at 95 °C for 15 minutes and centrifuged at 14,680 rpm for 10 minutes. Ten microliters of the supernatant were taken and transferred to a new microtube containing 90 µl of sterile molecular grade distilled water and stored at –20 °C until used. A Random Amplification of Polymorphic DNA (RAPD) was performed to confirm that the isolated strain not clones. Genetic profiles were performed using the following PCR conditions: 12.5 µl of Dream Taq Green PCR Master Mix (Thermo Fisher Scientific), 2.5 µl of primer M13 with a concentration of 20 µM (5′-CAGGGTGGCGGTTCT-3′) [16], 8.5 µl of miliQ water and 1.5 of DNA. The parameters of the thermocycler were: 95 °C for 10 min; 35 cycles of 94 °C for 1 min, 45 °C for 1 min and 72 °C for 2 min; and a final extension of 72 °C for 7 minutes. The amplicon was visualized using 1.5% agarose gel electrophoresis in Tris Acetate-EDTA buffer (TAE). The gel was stained with an ethidium bromide solution of 0.5 µg/ml for 30 minutes and washed with distilled water for another 30 minutes. The gel was photographed with a photodocumentation system (Gel Doc 2000 Bio-Rad, EEUU) and the band patterns were analyzed using the BioNumeric software (Applied Mathematics, Kortrijh, Belgium).

The 16S rRNA genes of the NTRH2 and NTRH6 strains were amplified and sequenced using the following methods: 15 µl of REDTaq® ReadyMix™ PCR Reaction Mix (Sigma), 2 µl forward primer (2 µM) (27F; 5′-GCCTGGGGAGTACGGCCGCA-3′), 2 µl of reverse primer (2 µM) (1522R; 5′-AAGGAGGTGATCCANCCRCA-3′), 9 µl of miliQ water and 2 µl of DNA. The thermocycler parameters were: 95 °C for 9 min; 35 cycles of 94 °C for 1 min, 56 °C for 1 min with 20 sec and 72 °C for 2 min; and a final extension of 72 °C for 7 min. The amplicon was extracted from a 1% agarose gel using the GeneJET DNA Cleanup Micro Kit (Thermo Scientific) using the manufacturer's specifications and sequenced (NUCLEUS Service, University of Salamanca). The sequences obtained were analyzed using the EzTaxon database.

### 2.3. Plant growth promotion mechanism

The ability of the strains to produce indole-3-acetic acid (IAA) was tested in JMM medium, as described by O'Hara et al. (1989) [17], using the agent of Salkowsky and quantified by spectrometry (550nm). The ability of strains to form biofilms was determined based on the method described by Fujishige et al. (2006) [18] in PVC microtiter plates. The absorbance was measured by a plate reader (Biochrom Asys UVM340) at 570 nm at 24, 48 and 72 hours.

Plate assays were also performed to determine whether the strains were capable of producing cellulose and cellulases. In the case of cellulose, 25 mg/L of Congo Red was added to the YMA medium, which binds to the β-1-4 linkages in cellulose, where cellulose producing colonies are stained red. To check for cellulocilitic activity, a double layer assay was performed as described by Mateos et al. (1992) [19]. In short, YMA medium plates were prepared and a layer of 1% CMC in 100 mM PCA and 0.5% agarose was overlaid onto the plates. To test for cellulose degradation, cellulases were inoculated with 5 µl (0.5 of optical density, 600 nm) of the corresponding bacterial suspension and incubated at 28 °C for 3 days.

### 2.4. *In vitro* and *in vivo* assays

To determine the nodulation capacity of the strains used in this study, *Trifolium rubens* L. seeds (from Thompson & Morgan, UK) were sterilized with 50% commercial bleach for 15 minutes and then washed 5 times with sterile distilled water. The seeds were pre-germinated on 1.5% water-agar plates, and then incubated at 28 °C for 2–3 days in complete darkness. Seedlings were placed on previously autoclaved filter papers in sterile test tubes containing Fahraeus medium [20]. Fifteen plants were used per treatment, which included: the uninoculated control and separate inoculations with NTRH2, NTRH6 and ATCC 14480. Each tube was inoculated with 1 ml of the corresponding bacterial suspension at an optical density of 0.5 (600 nm). The seedlings were incubated in a growth chamber at 22 °C at a relative humidity of 50–60% and with a photoperiod of 16 hours of light and 8 hours of darkness. Their growth (plant length and number of leaves) and nodulation effectiveness (number and color of nodules) were evaluated weekly. After 43 days, the plants were removed and the nodules were stored in formaldehyde (4%) until they were processed 24 hours later at the Tumor Bank of the Cancer Research Center of the University of Salamanca-CSIC. The nodules were first dehydrated through an ethanol series of 30% to 95% ETOH, and then cleared in xylol in a Thermo Shandon Excelsior processor. The material was then embedded in paraffin and sectioned into 2–3 µm sections using a HM 310 Microtome and placed over a microscope slide with a small amount of 3% bovine serum albumin (BSA) in PBS. The sections were deparaffinized using two xylol treatments, followed by treatments with absolute ethanol and then decreasing concentrations of ethanol and, finally water. The nodule tissue sections were stained with a solution of toluidine blue (0.01%) between 30 seconds to 1 minute. The microscope slides were rinsed with water, covered with a slide cover and observed under the light microscope and histologically analyzed.

Greenhouse assays were performed at the Spanish-Portuguese Agricultural Research Center (CIALE) in Salamanca, Spain. *Trifolium rubens* L. seeds were surface sterilized, pre-germinated and inoculated in the same manner as described above. Seedlings were transferred to a mixture of non-sterile peat-vermiculite (3:1). Two months later, 15 plants from each treatment were collected and their length, weight, chlorophyll content (Konica Minolta SPAD-502 PLUS) and the number and color of the nodules were analyzed. In addition, the plants were dried at 50 °C for 4 days to determine the dry weight of both shoots and roots. Following on, the aerial parts of the plants were dried and milled and processed by the Ionomic Service of the Soil and Applied Biology Center of Segura (CEBAS), CSIC (Spain) to determine the nitrogen contents.

The data were analyzed with ANOVA using Statview 5.0 software (SAS Institute Inc.) with statistically significant differences *P* < 0.05.

## 3. Results and Discussion

### 3.1. Isolation strain and genetic analysis

**Figure 1.**
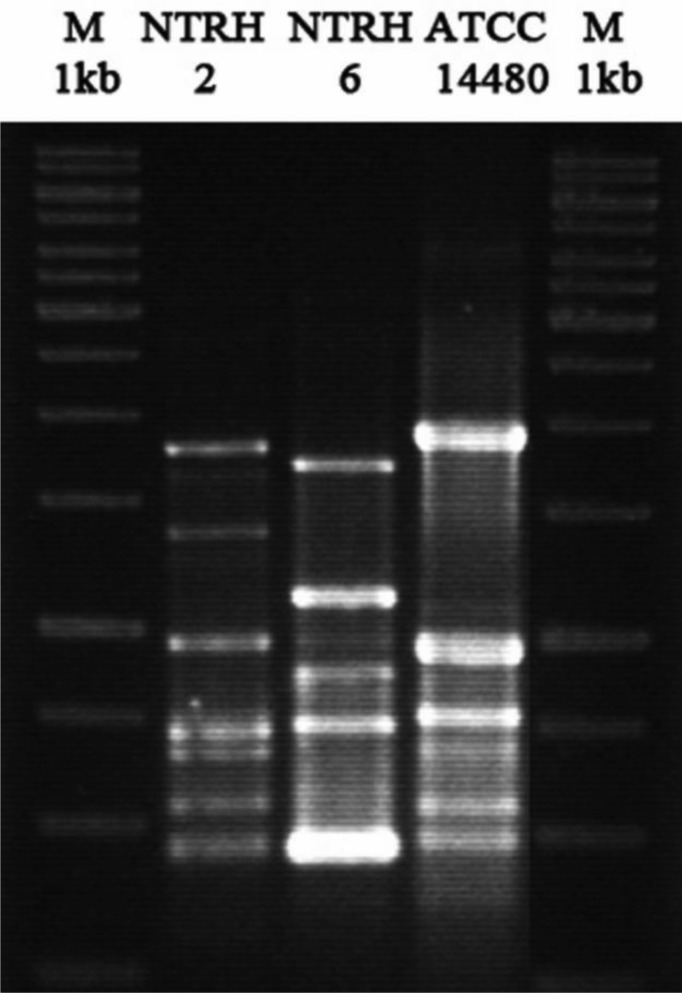
RAPD fingerprints of strains NTRH2, NTRH6 and *Rhizobium leguminosarum* bv. trifolii ATCC 14480 obtained using the M13 primer.

The bacterial colonies of two strains isolated from the nodules of *Trifolium repens* L. (NTRH2 and NTRH6) showed a morphology typical of rhizobial strains: white, convex and mucoid. The RAPD profiles determined that the strains NTRH2, NTRH6 and *Rhizobium leguminosarum* bv. trifolii ATCC 14480 had different genetic profiles (Figure 1), confirming that the three isolates were separate strains. The M13 universal primer is non-specific and amplifies diverse regions of the genome under defined conditions, producing different types of banding patterns. The genetic profiles that are generated have been used to study diversity within microbial communities [21],[22]. In addition, RAPD has also been applied to the study of rhizobial diversity [23],[24]. Hence, this technique allows the infraspecific diversity of microorganisms isolated from a nodule to be determined, where these microorganisms could either belong to different species or be different strains within the same species [25]. According to the 16S rRNA gene sequences obtained, it was possible to identify our strains as *Rhizobium* spp., which were shown to be very close to *R. anhuiense*, *R. gallicum*, *R. laguerreae*, *R. pisi, R. sophorae, R. acidisoli* and *R. fabae.*

### 3.2. *In vitro* analysis of plant growth promotion

The capacity of our strains to produce indole-3-acetic acid (IAA), a phytohormone produced by some PGPR strains which promotes plant growth, was tested. *In vitro* assays showed that all three strains, NTRH2, NTRH6 and ATCC 14480, were able to produce IAA. The strain with the best results was ATCC 14480, with an absorbance of 0.147, where strains NTRH6 and NTRH2 had an absorbance of 0.027 and 0.011, respectively. These results were quite significant, as IAA is capable of controlling several physiological processes such as the stimulation of growth and primary and secondary root elongation [26],[27]. Moreover, IAA increases root hair density which leads to greater nutrient and water absorption, all of which can help to improve plant development [28]. The production of IAA has been described in several rhizobia genera such as: *Rhizobium* [29]–[33], in addition, *Ensifer*, *Mesorhizobium*, *Bradyrhizobium*, *Allorhizobium* among others genera [34]. Therefore, the use of these strains to inoculate *T. rubens* L. plants could increase root formation which would in turn facilitate the absorption of nutrients, producing healthier and stronger plants.

In addition, the capacity of our strains to produce cellulose was analyzed. The assays showed that a red coloration was observed in the colonies 3 days after incubation, as Congo red was bound to the cellulose or other polysaccharide with β-1,4 bonds. By contrast, non-cellulose-producing colonies did not stain red and remained white. According to the results, the strain NTRH2 produced the largest amount of cellulose, followed by ATCC 14480; NTRH6 produced the least amount of cellulose. This type of exopolysaccharide may have the ability to produce biofilms, since cellulose can confer cohesion and elasticity [35], increasing root anchorage and allowing symbiotic relationships between PGPR and plants to be established.

Since it is known that some bacterial strains are capable of producing cellulose and other exopolysaccharides, the ability of our strains to produce biofilms was evaluated. Biofilms are a microbial community of cells that adhere to a surface, forming an extracellular matrix, [36] that can create a mechanism of resistance, involving one or more bacterial species. The high level of complexity of biofilms can provide metabolic advantages such as supporting abiotic and biotic factors, facilitating the assimilation of nutrients, among other advantages [37],[38]. Most microorganisms are able to form biofilms, which has allowed them to adapt to different microorganism communities within various types of niches [39]. The assay to test for the production of biofilms showed that all three strains were capable of producing biofilms *in vitro*. It was observed that NTRH2 had a lower capacity to produce biofilms during the first two days compared to NTRH6 and ATCC 14480 strains. However, at 72 hours all strains displayed a good capacity to form biofilms.

In addition, the strains ability to degrade carboxymethylcellulose, which would indicate cellulolytic activity, was tested. Cellulases play a very important role in the biosphere as they degrade cellulose, the most abundant carbon source on Earth. It has been reported that this enzyme is implicated in the degradation of non-crystalline cellulose. In rhizobia, cellulases are responsible for the “hole on the tip” (hot) phenotype, an aperture in the tip of the root hairs that allows symbiotic bacteria to enter inside the plant in a controlled way [19]. Strains NTRH2 and ATCC 14480 showed to be good producers of cellulases; strain NTRH6 was also able to produce cellulases although in smaller amounts.

### 3.3. *In vitro* plant growth assays

Once the potential of these strains to promote plant growth was characterized, their ability to nodulate *T. rubens* plants was tested. Also, the effects of the inoculum in early plant developmental stages were determined. It was found that 10 days after *T. rubens* seeds were inoculated with the various strains, the outer seed shells were already removed from the seedlings, unlike the control seedlings. Thus, in the inoculated treatments the seedlings had expelled their seed shells and had begun to develop cotyledons that were already showing signs of green coloration. At 15 days post inoculation, all of the inoculated strains already had their first true leaves. At this same developmental stage, the uninoculated control seedling only had two cotyledons. At 29 days post inoculation, the number of leaves and the lengths of the plants were considerably greater than those of the uninoculated control, and between 36 and 43 days post inoculation the development of nodules could already be observed. Therefore, these results indicate that the inoculation of the strains NRTH2, NTRH6 and ATCC 14480 on *T. rubens* L. seeds positively promotes the development of *T. rubens* L. seedling and subsequent nodulation.

**Figure 2.**
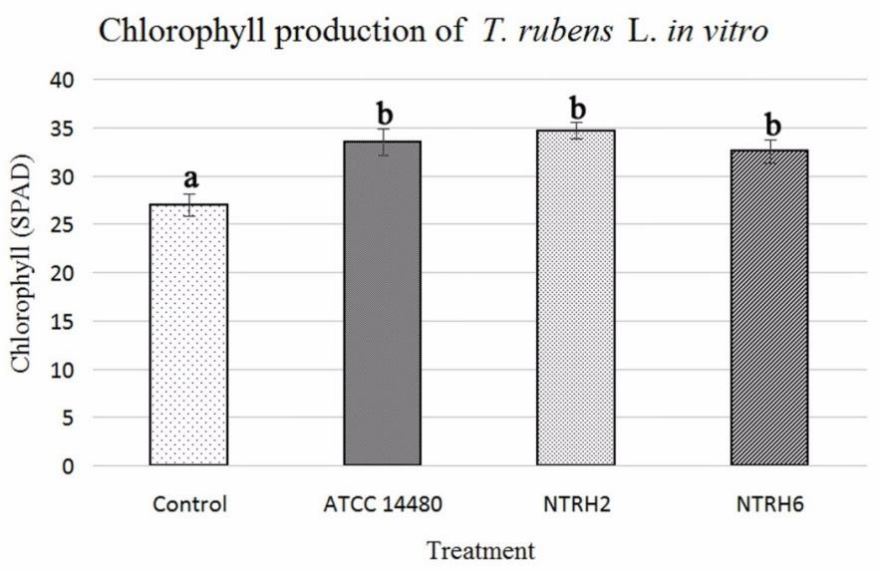
Measurement of chlorophyll contents in leaves of *T. rubens* L. plants grown *in vitro* at 43 days post-inoculation. The results with the same letter are not significantly different at *P* < 0.05.

In addition, the chlorophyll content of the inoculated plants was measured, but no statistically significant differences were detected among inoculants. However, all inoculated plants showed higher chlorophyll concentrations as compared to the negative control group, as shown in Figure 2. The nutritional status of a crop can be evaluated by measuring the chlorophyll content within leaves, and the lack of nitrogen can affect chlorophyll content [40]. Hence, chlorophyll can be used a way to monitor the availability of N [41]. This idea is supported by the studies of Mendoza et al. (1998) [42] and Sainz-Rozas & Echeverria (1998) [40] who show that there is a high correlation between chlorophyll content and nitrogen content in a plant.

**Figure 3.**
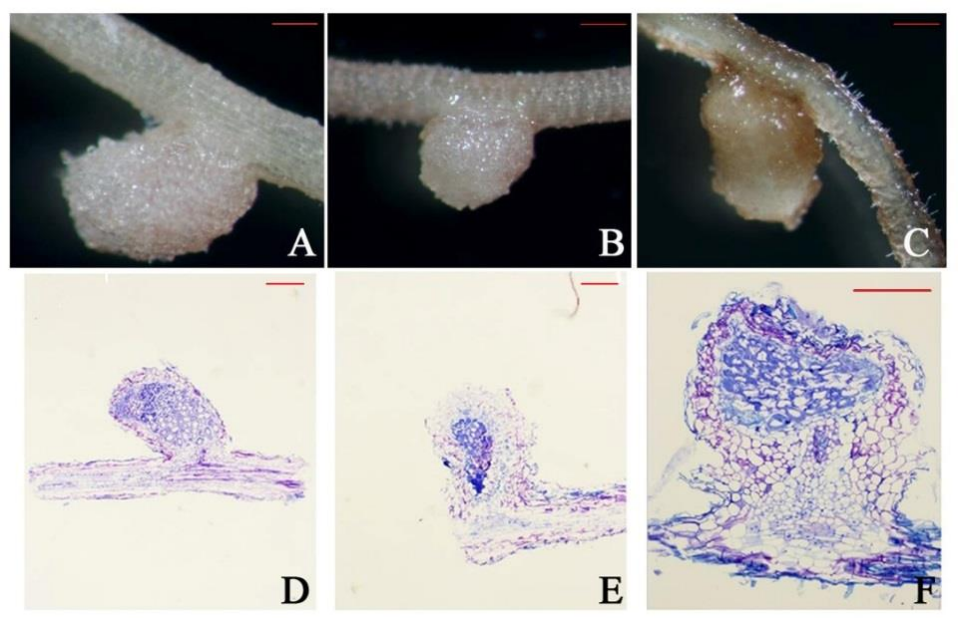
Nodules formed in *T. rubens* L. by the strains isolated in this study. A, B and C show the external nodule formation formed by strain NTRH2 (A), NTRH6 (B) and ATCC 14480 (C). D, E and F are sections of embedded nodules stained with Toluidine Blue: NTRH2 (D), NTRH6 (E) and ATCC 14480 (F). Bar: 200 µm.

All strains showed the presence of nodules (Figure 3). In all cases, the nodules had a pink color due to the presence of leghaemoglobin, an enzyme responsible for sequestering oxygen molecules. Therefore, it can be concluded that the conditions of the nodule were suitable for the proper functioning of nitrogenase, and that the nodules were most probably efficient. Upon analyzing the histological cuts of nodules stained with Toluidine Blue (Figure 3), the bacteroids could be visualized within the blue stained areas. Thus, these areas show that all three strains have the capacity to fix nitrogen. Furthermore, the three strains showed no significant differences with respect to number of nodules in the *in vitro* assays.

### 3.4. *In vivo* plant growth assays

**Figure 4.**
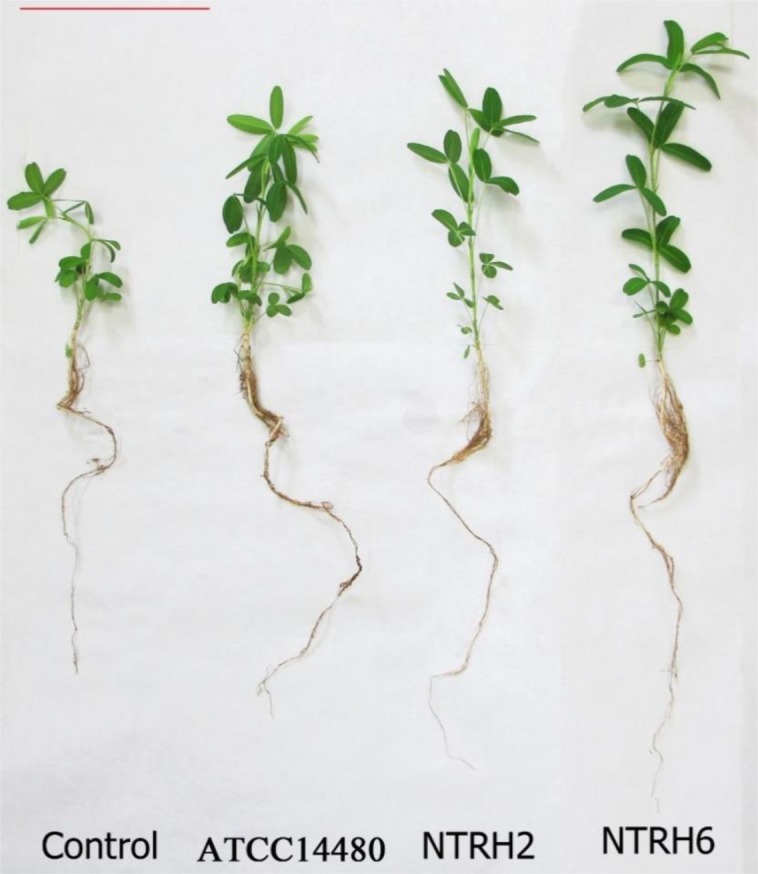
Representative *T. rubens* L. inoculated plants at 67 days post inoculation under greenhouse conditions. Bar: 10 cm.

Among the inoculated plants, differences could already be observed at 18 days post inoculation with respect to the control. Also, at 67 days, differences in plant lengths were also observed, where the inoculated plants exhibited a greater number of leaves and aerial and radicular elongation, Figure 4.

Regarding plant growth, one of the notable characteristic was that the inoculate plants showed a better root system, which in turn would provide the plant with an improved ability to absorb nutrients and necessary elements and facilitate water catchment [43]. Also, the measurements of both root dry weight and chlorophyll were significantly greater for the inoculated plants than in the control. In the case of chlorophyll, reconfirmed as already seen in the *in vitro* system. In addition, higher values were obtained in all treatments on the control (2.17 g/100 g), upon analyzing the nitrogen content of *T. rubens* L. plants; showing the highest values of ATCC 14480 (2.56 g/100 g), NTRH2 (2.75 g/100 g) and NRTH6 (3.16 g/100 g) strains. These results agree with the measurements of chlorophyll in SPAD units, since the treatment with the greater amount of chlorophyll proved to be the one that showed the greater amount of nitrogen. In addition, the plants with the lowest amount of chlorophyll (negative control) also had the lowest amount of nitrogen. Therefore, since statistically significant results were obtained for all treatments as compared to the control, it can be said that there was a higher level of nitrogen fixation, results that are in line with other studies [40],[41].

Regarding root dry weight, it was found that all treatments doubled the root dry weight of uninoculated control plants (Table 1). Also, the shoot dry weights of all inoculated plants had better weights as compared to the negative control. Nodules were found in all of the treatments, including the uninoculated control, since the substrate used was not sterile. Furthermore, the numbers of nodules among the control and the NTRH2 and NRTH6 strains showed no significant differences; however, ATCC 14480 had the highest number of nodules. Although it must be noted that the quantity of nodules is not equivalent to better plant development. Thus, even though the control had a large number of nodules they were smaller in size and whitish, where the nodules of the plants treated with the various strains were larger in size and were pink in color, suggesting that the symbiotic relationship was more efficient in these nodules.

**Table 1.** Measurements of chlorophyll leaf content, number of nodules and lengths and weights of *T. rubens* L. inoculated plants.

Strain	Uninoculated control	NTRH2	NTRH6	ATCC 14480
Chlorophyll (SPAD)	28.72 ± 0.59^a^	34.03 ± 0.47^b^	32.15 ± 0.43^b^	32.23 ± 0.75^b^
Number of nodules	97.38 ± 8.53^a^	99.75 ± 6.32^a^	114.13 ± 6.03^a^	117.75 ± 5.89^b^
Shoot length (cm)	13.51 ± 1.28^a^	17.61 ± 0.58^b^	20.39 ± 0.82^b^	17.69 ± 1.25^b^
Root length (cm)	24.71 ± 1.66^a^	31.90 ± 1.37^b^	34.94 ± 1.59^c,b^	30.08 ± 1.97^b^
Wet shoot weight (g)	0.59 ± 0.09^a^	1.24 ± 0.21^b^	1.71 ± 0.27^b^	1.34 ± 0.15^b^
Dry shoot weight (g)	0.15 ± 0.03^a^	0.41 ± 0.09^b^	0.34 ± 0.06^b^	0.27 ± 0.03^b^
Wet root weight (g)	0.54 ± 0.10^a^	1.14 ± 0.14^b^	1.30 ± 0.13^b^	1.20 ± 0.12^b^
Dry root weight (g)	0.08 ± 0.02^a^	0.19 ± 0.03^b^	0.18 ± 0.02^b^	0.16 ± 0.02^b^

Values followed by the same letter are not significantly different at *P* < 0.05.

Once the potential of these strains isolated from nodules not belonging to *T. rubens* L. as a future projection we intend to evaluate their competitiveness on the native populations of these plants since their inoculation could modify their community composition and/or quantity [44], either positively (good fixation of nitrogen, occupy a niche that could be occupied by some pathogen, etc.) or negatively (competing adversely against native rhizobia better nitrogen fixers that our strains). To date, the studies on *Trifolium rubens* L. have been quite scarce, therefore our results add to the knowledge regarding this species.

## 4. Conclusions

The strains NTRH2, NTRH6 and ATCC 14480 belong to the genus *Rhizobium* and are able to nodulate *Trifolium rubens* L. plants, and enhance their growth and development without creating any adverse effects. This is achieved by increasing overall shoot and root growth as compared to uninoculated plants. Thus, these strains could be used in an ecological way for the conservation of this species, classified as Preferential Attention in the Autonomous Community of Castile and Leon in Spain. These strains could also be applied as biofertilizers on soils poor in nitrogen and on the areas where these plants grow, with the aim of increasing the number of populations. In sum, this study highlights the potential that some microorganisms have in improving the conservation status of plants classified as vulnerable, endangered and even critically endangered.
